# *catena*-Poly[[(1,10-phenanthroline-κ^2^ *N*,*N*′)copper(II)]-μ-2,2′-iminodibenzoato-κ^4^ *O*,*O*′:*O*′′,*O*′′′]

**DOI:** 10.1107/S1600536813009203

**Published:** 2013-04-13

**Authors:** Consuelo Yuste-Vivas, Joana T. Coutinho, Laura C. J. Pereira, Manuela Ramos Silva

**Affiliations:** a CEMDRX, Physics Department, University of Coimbra, P-3004-516 Coimbra, Portugal; b Solid State Group UCQR, IST/ITN, Universidade Técnica de Lisboa, Estrada Nacional 10, 2686-953 Sacavém, Portugal

## Abstract

The structure of the title compound, [Cu(C_14_H_9_NO_4_)(C_12_H_8_N_2_)]_*n*_, consists of zigzag polymeric chains along the *c* axis. The asymmetric unit contains one Cu^II^ atom which is coordinated by one 2,2′-imino­dibenzoate ligand and a one phenanthroline unit. Two intra­molecular N—H⋯O hydrogen bonds occur. The supra­molecular structure is characterized by weak C—H⋯O hydrogen bonds and π–π stacking inter­actions, forming a three-dimensional supramolecular network. The shortest centroid–centroid distances between neighbouring phenanthroline aromatic rings and 2,2′-imino­dibenzoate rings are 3.684 (1) and 3.640 Å, respectively. The shortest intra­chain Cu⋯Cu distance is 7.2885 (9) and the shortest Cu⋯Cu distance between Cu atoms in different chains is 7.1103 (6) Å.

## Related literature

For general background to Cu^II^ low-dimensional polynuclear magnetic materials, see: Fabelo *et al.* (2009 ▶); Martins *et al.* (2008*a* ▶,*b* ▶); Silva *et al.* (2001 ▶); Yuste *et al.* (2007 ▶, 2008 ▶). For structural and coordination information for 2,2′-iminodi­benzoic acid, see: Field & Venkataraman (2002 ▶); Gao *et al.* (2009 ▶); Lin *et al.* (2006 ▶).
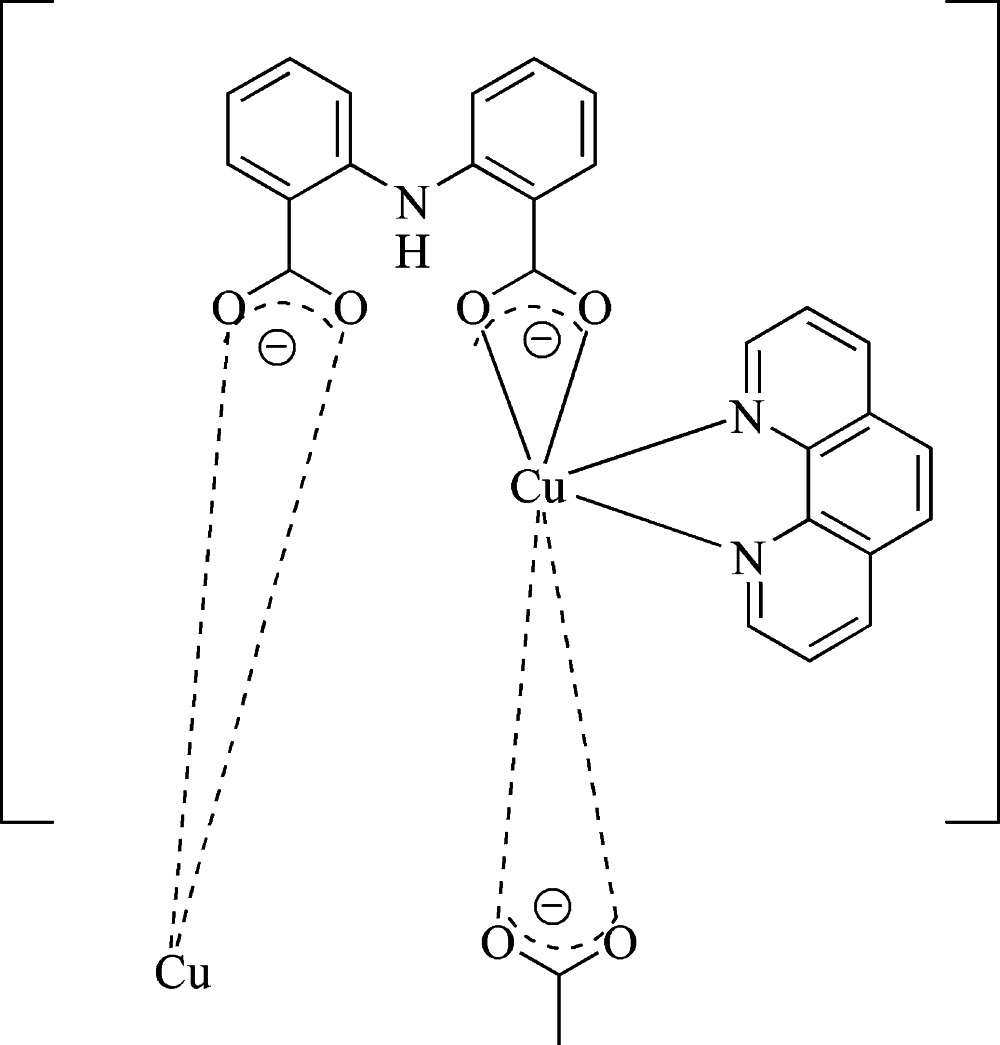


## Experimental

### 

#### Crystal data

[Cu(C_14_H_9_NO_4_)(C_12_H_8_N_2_)]
*M* *_r_* = 498.98
Monoclinic, 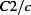

*a* = 31.7536 (6) Å
*b* = 9.8492 (2) Å
*c* = 14.4865 (3) Å
β = 113.222 (1)°
*V* = 4163.56 (14) Å^3^
*Z* = 8
Mo *K*α radiation
μ = 1.09 mm^−1^
*T* = 293 K
0.1 × 0.08 × 0.07 mm

#### Data collection

Bruker APEXII CCD area-detector diffractometer
Absorption correction: multi-scan (*SADABS*; Sheldrick, 2003 ▶) *T*_min_ = 0.898, *T*_max_ = 0.971
36778 measured reflections
3976 independent reflections
2900 reflections with *I* > 2σ(*I*)
*R*_int_ = 0.061

#### Refinement

*R*[*F* ^2^ > 2σ(*F* ^2^)] = 0.035
*wR*(*F* ^2^) = 0.089
*S* = 1.02
3976 reflections
307 parameters
H-atom parameters constrained
Δρ_max_ = 0.29 e Å^−3^
Δρ_min_ = −0.40 e Å^−3^

### 

Data collection: *APEX2* (Bruker, 2003 ▶); cell refinement: *SAINT* (Bruker, 2003 ▶); data reduction: *SAINT*; program(s) used to solve structure: *SHELXS97* (Sheldrick, 2008 ▶); program(s) used to refine structure: *SHELXL97* (Sheldrick, 2008 ▶); molecular graphics: *DIAMOND* (Brandenburg, 2006 ▶); software used to prepare material for publication: *WinGX* publication routines (Farrugia, 2012 ▶).

## Supplementary Material

Crystal structure: contains datablock(s) I, global. DOI: 10.1107/S1600536813009203/bt6890sup1.cif

Structure factors: contains datablock(s) I. DOI: 10.1107/S1600536813009203/bt6890Isup2.hkl

Additional supplementary materials:  crystallographic information; 3D view; checkCIF report

## Figures and Tables

**Table 1:** Hydrogen-bond geometry (Å, °)

*D*—H⋯*A*	*D*—H	H⋯*A*	*D*⋯*A*	*D*—H⋯*A*
N1—H1⋯O2	0.86	2.07	2.708 (5)	131
N1—H1⋯O3	0.86	2.06	2.701 (5)	130
C17—H17⋯O2^i^	0.93	2.55	3.308 (4)	139
C23—H23⋯O3^ii^	0.93	2.38	3.185 (4)	145

Symmetry codes: (i) 
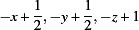
; (ii) 
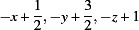
.

**Fig. 1.**
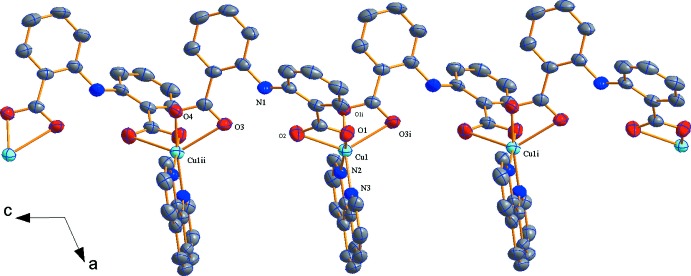
: The molecular structure of the title compound, showing the atom-labelling scheme and displacement ellipsoids drawn at the 50% probability level. [Symmetry codes: (i) x, 1 - y, 1/2 - z; (ii) x, 1 - y, 1/2 + z.]

**Fig. 2.**
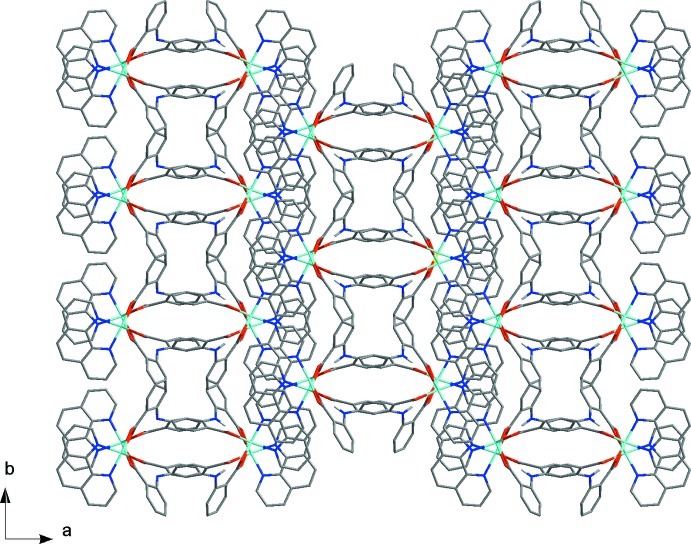
: View of the crystal packing of the title compound, projected along c.

**Fig. 3.**
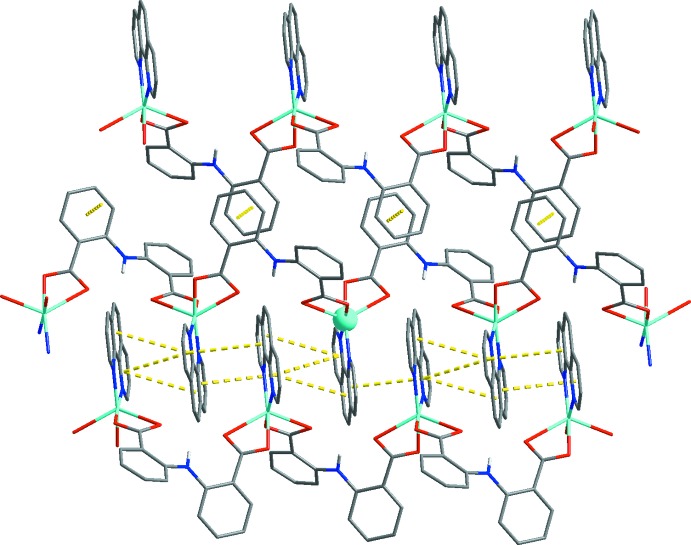
: A view showing part of the three-dimensional supramolecular network linked by weak π-π stacking interactions (yellow dotted lines).

## supplementary crystallographic information

### Comment

This work is part of a project of synthesizing low dimensional polynuclear magnetic materials with Copper(II) and oxygen-donors bridging ligands (Fabelo *et al.*, 2009; Martins *et al.*, 2008*a*; Martins *et al.*, 2008*b*; Silva *et al.*, 2001; Yuste *et al.*, 2007, 2008).

The target of this work is the use of an aromatic dicarboxylic acid, such as the 2,2'-iminodibenzoic acid, (H_2_IDC) and another quelate, known as ''coligand'' that will block some coordination positions of the Copper(II) metal ion, modulating the dimensionality of the resulting compound. (Gao *et al.*, 2009; Lin *et al.*, 2006; Yuste *et al.*, 2008).

The structure of this compound consists of neutral chains of formula [Cu(C_14_H_9_NO_4_)(C_12_H_8_N_2_)]*_n_*, growing along the *c*-axis, in a zigzag mode, where the 2,2'-iminodibenzoate (IDC^2-^) units act as linkers between two Cu(II) ions, in a bis-bidentate mode, and the phenanthroline molecules are placed out-of-chain. The whole compound adopts a three dimensional supramolecular structure by weak π-π stacking. The shortest intra- and interchain copper···copper distances are 7.2885 (9) Å [Cu1···Cu1^i^; (i) = *x*, 1 - *y*, -1/2 + *z*] and 7.1103 (6) Å [Cu1···Cu1^v^; (v) = 1/2 - *x*, 1/2 - *y*, 1 - *z*], respectively.

The Copper(II) ion shows a distorted octahedral environment, CuN_2_O_4_, due to the Jahn-Teller effect. The equatorial positions are occupied by the two nitrogen atoms from the phenanthroline ligand, [N1 and N2], and two oxygen atoms [O1 and O4], from two different carboxylate units of the IDC^2-^ ligand, varying the distances in a very narrow range of [1.940–2.026 Å]. Another two oxygen atoms [O2 and O3], with bond length values 2.618 (2) and 2.438 (3) Å respectively, are placed in the axial positions. The 2,2'-iminodibenzoate links two neighboring Copper(II) metal ions, being the bite angle 55.19 (11)° [O1—Cu1—O2] and 58.92 (11)° [O3—Cu1^ii^—O4]. [(ii) = *x*, -*y*, -1/2 + *z*]. The ligand is not planar, with a maximum deviation of 1.472 (5) Å for C10 from the mean plane, being the dihedral angle between the two aromatic rings 52.25 (3)°, which is greater than those already reported (Field *et al.*, 2002; Gao *et al.*, 2009).

Intramolecular hydrogen bond interactions exist inside the 2,2'-iminodibenzoate unit between the nitrogen atom [N1] from the amino group and two oxygen atoms [O2 and O3] from the carboxylate groups. The intermolecular π-π stacking interaction exists in between two aromatic rings of two neighbor phenantrolines and also between the aromatic rings of two neighbor 2,2'-iminodibenzoate moieties. These weak π-π interactions, stabilize the crystal structure of the complex. The shortest distances 'centroid-to-centroid' between neighbor aromatic ring of two phenantrolines and two neighbor 2,2'-iminodibenzoate are 3.684 (1) and 3.640 Å respectively.

### Experimental

All the reagents, phenanthroline, 2,2'-Iminodibenzoic acid, and the metallic salt Cu(NO_3_)_2_.3H_2_O, were purchased from commercial sources and used as received with no further purifications.

An aqueous solution containing Cu(NO_3_)_2_.3H_2_O (1 mmol, 0.0242 g), Iminodibenzoic acid (1 mmol, 0.0257 g) and phenanthroline, ((2 mmol, 0.0361 g), was stirred during 30 minutes and placed in a 25 mL Teflon-lined autoclave and then heated at 120°C during 48 h. Dark green crystals were obtained by filtration, washed with water and dried in air. Ca. 36% yield based on Cu.

### Refinement

All H atoms could be located in a difference Fourier synthesis but were placed in calculated positions and refined as riding on their parent atoms, using *SHELXL* (Sheldrick, 2008) defaults.

### Crystal data

**Table:**

[Cu(C_14_H_9_NO_4_)(C_12_H_8_N_2_)]	*F*(000) = 2040
*M**_r_* = 498.98	*D*_x_ = 1.592 Mg m^−^^3^
Monoclinic, *C*2/*c*	Mo *K*α radiation, λ = 0.71073 Å
Hall symbol: -C 2yc	Cell parameters from 6606 reflections
*a* = 31.7536 (6) Å	θ = 2.2–20.8°
*b* = 9.8492 (2) Å	µ = 1.09 mm^−^^1^
*c* = 14.4865 (3) Å	*T* = 293 K
β = 113.222 (1)°	Blocks, green
*V* = 4163.56 (14) Å^3^	0.1 × 0.08 × 0.07 mm
*Z* = 8	

### Data collection

**Table:**

Bruker APEXII CCD area-detector diffractometer	3976 independent reflections
Radiation source: fine-focus sealed tube	2900 reflections with *I* > 2σ(*I*)
Graphite monochromator	*R*_int_ = 0.061
φ and ω scans	θ_max_ = 25.8°, θ_min_ = 2.8°
Absorption correction: multi-scan (*SADABS*; Sheldrick, 2003)	*h* = −38→38
*T*_min_ = 0.898, *T*_max_ = 0.971	*k* = −12→12
36778 measured reflections	*l* = −17→17

### Refinement

**Table:**

Refinement on *F*^2^	Primary atom site location: structure-invariant direct methods
Least-squares matrix: full	Secondary atom site location: difference Fourier map
*R*[*F*^2^ > 2σ(*F*^2^)] = 0.035	Hydrogen site location: inferred from neighbouring sites
*wR*(*F*^2^) = 0.089	H-atom parameters constrained
*S* = 1.02	*w* = 1/[σ^2^(*F*_o_^2^) + (0.037*P*)^2^ + 5.032*P*] where *P* = (*F*_o_^2^ + 2*F*_c_^2^)/3
3976 reflections	(Δ/σ)_max_ < 0.001
307 parameters	Δρ_max_ = 0.29 e Å^−^^3^
0 restraints	Δρ_min_ = −0.40 e Å^−^^3^

### Special details

**Table:**

Geometry. All e.s.d.'s (except the e.s.d. in the dihedral angle between two l.s. planes) are estimated using the full covariance matrix. The cell e.s.d.'s are taken into account individually in the estimation of e.s.d.'s in distances, angles and torsion angles; correlations between e.s.d.'s in cell parameters are only used when they are defined by crystal symmetry. An approximate (isotropic) treatment of cell e.s.d.'s is used for estimating e.s.d.'s involving l.s. planes.
Refinement. Refinement of *F*^2^ against ALL reflections. The weighted *R*-factor *wR* and goodness of fit *S* are based on *F*^2^, conventional *R*-factors *R* are based on *F*, with *F* set to zero for negative *F*^2^. The threshold expression of *F*^2^ > σ(*F*^2^) is used only for calculating *R*-factors(gt) *etc*. and is not relevant to the choice of reflections for refinement. *R*-factors based on *F*^2^ are statistically about twice as large as those based on *F*, and *R*- factors based on ALL data will be even larger.

### Fractional atomic coordinates and isotropic or equivalent isotropic displacement parameters (Å^2^)

**Table:**

	*x*	*y*	*z*	*U*_iso_*/*U*_eq_	
Cu1	0.172485 (11)	0.45882 (4)	0.30778 (3)	0.03710 (12)	
N1	0.07823 (8)	0.7287 (3)	0.46195 (18)	0.0466 (6)	
H1	0.1021	0.6781	0.4882	0.056*	
N2	0.20508 (7)	0.2788 (2)	0.35164 (16)	0.0361 (5)	
N3	0.23714 (7)	0.5223 (2)	0.34707 (17)	0.0370 (5)	
O4	0.11136 (6)	0.6223 (2)	0.75794 (15)	0.0448 (5)	
O1	0.14757 (7)	0.6413 (2)	0.27927 (16)	0.0479 (5)	
O2	0.14659 (7)	0.6025 (2)	0.42821 (15)	0.0495 (5)	
O3	0.13160 (6)	0.5939 (2)	0.63028 (15)	0.0441 (5)	
C1	0.13485 (9)	0.6710 (3)	0.3501 (2)	0.0387 (7)	
C2	0.10555 (8)	0.7946 (3)	0.3344 (2)	0.0361 (7)	
C3	0.10490 (9)	0.8884 (3)	0.2622 (2)	0.0444 (7)	
H3	0.1217	0.8703	0.2237	0.053*	
C4	0.08029 (11)	1.0071 (3)	0.2458 (3)	0.0539 (9)	
H4	0.0798	1.0670	0.1957	0.065*	
C5	0.05662 (10)	1.0354 (3)	0.3043 (3)	0.0560 (9)	
H5	0.0414	1.1179	0.2967	0.067*	
C6	0.05500 (10)	0.9437 (3)	0.3744 (3)	0.0528 (8)	
H6	0.0379	0.9641	0.4120	0.063*	
C7	0.07871 (9)	0.8197 (3)	0.3902 (2)	0.0384 (7)	
C8	0.04358 (9)	0.7090 (3)	0.4972 (2)	0.0414 (7)	
C9	−0.00235 (10)	0.7313 (4)	0.4351 (3)	0.0537 (9)	
H9	−0.0099	0.7659	0.3708	0.064*	
C10	−0.03645 (10)	0.7024 (4)	0.4680 (3)	0.0603 (10)	
H10	−0.0669	0.7152	0.4249	0.072*	
C11	−0.02635 (10)	0.6550 (4)	0.5636 (3)	0.0605 (10)	
H11	−0.0497	0.6372	0.5855	0.073*	
C12	0.01899 (10)	0.6343 (3)	0.6269 (3)	0.0492 (8)	
H12	0.0262	0.6032	0.6919	0.059*	
C13	0.05402 (9)	0.6595 (3)	0.5942 (2)	0.0383 (7)	
C14	0.10206 (9)	0.6238 (3)	0.6641 (2)	0.0381 (7)	
C15	0.18760 (10)	0.1574 (3)	0.3531 (2)	0.0440 (7)	
H15	0.1561	0.1494	0.3341	0.053*	
C16	0.21466 (11)	0.0407 (3)	0.3820 (2)	0.0502 (8)	
H16	0.2012	−0.0432	0.3822	0.060*	
C17	0.26097 (11)	0.0504 (3)	0.4102 (2)	0.0500 (8)	
H17	0.2793	−0.0266	0.4299	0.060*	
C18	0.28067 (10)	0.1772 (3)	0.4090 (2)	0.0421 (7)	
C19	0.32883 (10)	0.2002 (4)	0.4383 (2)	0.0519 (8)	
H19	0.3491	0.1273	0.4580	0.062*	
C20	0.34496 (10)	0.3263 (4)	0.4376 (2)	0.0515 (8)	
H20	0.3764	0.3388	0.4582	0.062*	
C21	0.31555 (9)	0.4409 (3)	0.4063 (2)	0.0409 (7)	
C22	0.32977 (11)	0.5755 (4)	0.4042 (2)	0.0516 (9)	
H22	0.3607	0.5948	0.4229	0.062*	
C23	0.29850 (11)	0.6773 (3)	0.3750 (2)	0.0523 (8)	
H23	0.3080	0.7664	0.3737	0.063*	
C24	0.25214 (10)	0.6482 (3)	0.3469 (2)	0.0454 (7)	
H24	0.2311	0.7190	0.3274	0.055*	
C25	0.26837 (9)	0.4207 (3)	0.3767 (2)	0.0357 (6)	
C26	0.25100 (9)	0.2879 (3)	0.3789 (2)	0.0351 (6)	

### Atomic displacement parameters (Å^2^)

**Table:**

	*U*^11^	*U*^22^	*U*^33^	*U*^12^	*U*^13^	*U*^23^
Cu1	0.03325 (18)	0.0380 (2)	0.0386 (2)	0.00164 (16)	0.01251 (14)	−0.00015 (17)
N1	0.0341 (13)	0.0582 (17)	0.0465 (16)	0.0182 (12)	0.0148 (11)	0.0099 (13)
N2	0.0334 (12)	0.0379 (14)	0.0331 (13)	−0.0015 (10)	0.0091 (10)	−0.0031 (11)
N3	0.0373 (12)	0.0364 (14)	0.0364 (13)	−0.0009 (11)	0.0137 (10)	−0.0022 (11)
O4	0.0373 (10)	0.0534 (13)	0.0395 (12)	0.0011 (10)	0.0106 (9)	−0.0050 (10)
O1	0.0541 (12)	0.0437 (12)	0.0529 (13)	0.0095 (10)	0.0286 (11)	0.0050 (11)
O2	0.0522 (12)	0.0514 (13)	0.0428 (13)	0.0222 (10)	0.0166 (10)	0.0085 (11)
O3	0.0325 (10)	0.0517 (13)	0.0484 (13)	0.0064 (9)	0.0162 (9)	0.0097 (10)
C1	0.0311 (14)	0.0354 (16)	0.0434 (18)	0.0016 (12)	0.0081 (13)	−0.0023 (14)
C2	0.0276 (13)	0.0328 (16)	0.0384 (16)	0.0010 (11)	0.0029 (12)	−0.0023 (13)
C3	0.0379 (15)	0.0434 (18)	0.0443 (18)	−0.0045 (14)	0.0081 (13)	−0.0003 (15)
C4	0.0439 (17)	0.0402 (18)	0.063 (2)	−0.0025 (14)	0.0055 (16)	0.0098 (16)
C5	0.0418 (17)	0.0348 (18)	0.071 (2)	0.0068 (15)	−0.0001 (16)	0.0034 (18)
C6	0.0398 (16)	0.051 (2)	0.059 (2)	0.0143 (15)	0.0105 (15)	−0.0053 (17)
C7	0.0285 (14)	0.0407 (17)	0.0370 (17)	0.0064 (12)	0.0033 (12)	0.0004 (14)
C8	0.0330 (15)	0.0418 (17)	0.0445 (18)	0.0066 (13)	0.0101 (13)	−0.0051 (14)
C9	0.0355 (16)	0.067 (2)	0.052 (2)	0.0124 (15)	0.0096 (14)	−0.0008 (17)
C10	0.0299 (16)	0.072 (3)	0.069 (3)	0.0086 (16)	0.0089 (16)	−0.005 (2)
C11	0.0356 (17)	0.068 (2)	0.083 (3)	−0.0017 (16)	0.0282 (17)	−0.006 (2)
C12	0.0401 (16)	0.052 (2)	0.056 (2)	−0.0007 (15)	0.0197 (15)	−0.0052 (16)
C13	0.0289 (14)	0.0340 (16)	0.0484 (18)	0.0012 (12)	0.0113 (12)	−0.0069 (14)
C14	0.0334 (15)	0.0320 (16)	0.0460 (19)	−0.0047 (12)	0.0125 (13)	−0.0006 (14)
C15	0.0420 (16)	0.0425 (19)	0.0415 (18)	−0.0063 (14)	0.0100 (13)	−0.0033 (14)
C16	0.064 (2)	0.0330 (17)	0.0458 (18)	−0.0072 (16)	0.0138 (15)	−0.0010 (15)
C17	0.060 (2)	0.0400 (18)	0.0423 (18)	0.0115 (16)	0.0119 (15)	−0.0002 (15)
C18	0.0449 (16)	0.0439 (18)	0.0341 (17)	0.0076 (14)	0.0120 (13)	−0.0006 (14)
C19	0.0391 (17)	0.064 (2)	0.050 (2)	0.0151 (16)	0.0141 (14)	0.0007 (17)
C20	0.0322 (15)	0.073 (2)	0.050 (2)	0.0047 (16)	0.0167 (14)	−0.0047 (18)
C21	0.0343 (14)	0.056 (2)	0.0358 (16)	−0.0040 (14)	0.0170 (12)	−0.0041 (15)
C22	0.0399 (17)	0.070 (2)	0.048 (2)	−0.0164 (16)	0.0203 (14)	−0.0093 (17)
C23	0.060 (2)	0.050 (2)	0.051 (2)	−0.0174 (17)	0.0258 (16)	−0.0057 (16)
C24	0.0505 (18)	0.0392 (18)	0.0472 (19)	−0.0048 (14)	0.0200 (15)	−0.0043 (15)
C25	0.0356 (14)	0.0425 (17)	0.0295 (15)	−0.0012 (12)	0.0135 (12)	−0.0031 (12)
C26	0.0361 (14)	0.0399 (16)	0.0284 (15)	0.0021 (12)	0.0117 (12)	−0.0006 (13)

### Geometric parameters (Å, º)

**Table:**

Cu1—O1	1.942 (2)	C8—C13	1.397 (4)
Cu1—O4^i^	1.9548 (19)	C9—C10	1.375 (4)
Cu1—N3	2.002 (2)	C9—H9	0.9300
Cu1—N2	2.025 (2)	C10—C11	1.374 (5)
Cu1—O3^i^	2.4360 (19)	C10—H10	0.9300
Cu1—C14^i^	2.515 (3)	C11—C12	1.383 (4)
N1—C7	1.377 (4)	C11—H11	0.9300
N1—C8	1.398 (4)	C12—C13	1.392 (4)
N1—H1	0.8600	C12—H12	0.9300
N2—C15	1.322 (4)	C13—C14	1.503 (4)
N2—C26	1.355 (3)	C14—Cu1^ii^	2.515 (3)
N3—C24	1.329 (4)	C15—C16	1.397 (4)
N3—C25	1.354 (3)	C15—H15	0.9300
O4—C14	1.272 (3)	C16—C17	1.366 (4)
O4—Cu1^ii^	1.9548 (19)	C16—H16	0.9300
O1—C1	1.276 (3)	C17—C18	1.399 (4)
O2—C1	1.241 (3)	C17—H17	0.9300
O3—C14	1.253 (3)	C18—C26	1.393 (4)
O3—Cu1^ii^	2.4360 (19)	C18—C19	1.435 (4)
C1—C2	1.494 (4)	C19—C20	1.345 (5)
C2—C3	1.389 (4)	C19—H19	0.9300
C2—C7	1.409 (4)	C20—C21	1.421 (4)
C3—C4	1.373 (4)	C20—H20	0.9300
C3—H3	0.9300	C21—C25	1.400 (4)
C4—C5	1.366 (5)	C21—C22	1.404 (4)
C4—H4	0.9300	C22—C23	1.357 (4)
C5—C6	1.374 (5)	C22—H22	0.9300
C5—H5	0.9300	C23—C24	1.394 (4)
C6—C7	1.406 (4)	C23—H23	0.9300
C6—H6	0.9300	C24—H24	0.9300
C8—C9	1.397 (4)	C25—C26	1.425 (4)
			
O1—Cu1—O4^i^	92.16 (9)	C11—C10—C9	121.2 (3)
O1—Cu1—N3	93.31 (9)	C11—C10—H10	119.4
O4^i^—Cu1—N3	171.94 (9)	C9—C10—H10	119.4
O1—Cu1—N2	172.92 (9)	C10—C11—C12	119.1 (3)
O4^i^—Cu1—N2	93.93 (9)	C10—C11—H11	120.5
N3—Cu1—N2	81.03 (9)	C12—C11—H11	120.5
O1—Cu1—O3^i^	88.28 (8)	C11—C12—C13	120.7 (3)
O4^i^—Cu1—O3^i^	58.99 (7)	C11—C12—H12	119.6
N3—Cu1—O3^i^	115.25 (8)	C13—C12—H12	119.6
N2—Cu1—O3^i^	97.95 (8)	C12—C13—C8	120.0 (3)
O1—Cu1—C14^i^	88.22 (9)	C12—C13—C14	117.6 (3)
O4^i^—Cu1—C14^i^	29.85 (8)	C8—C13—C14	122.3 (2)
N3—Cu1—C14^i^	144.48 (9)	O3—C14—O4	121.4 (2)
N2—Cu1—C14^i^	98.86 (9)	O3—C14—C13	120.6 (3)
O3^i^—Cu1—C14^i^	29.27 (8)	O4—C14—C13	118.0 (2)
C7—N1—C8	127.7 (2)	O3—C14—Cu1^ii^	71.87 (15)
C7—N1—H1	116.1	O4—C14—Cu1^ii^	49.89 (13)
C8—N1—H1	116.1	C13—C14—Cu1^ii^	165.6 (2)
C15—N2—C26	117.7 (2)	N2—C15—C16	122.5 (3)
C15—N2—Cu1	129.13 (19)	N2—C15—H15	118.8
C26—N2—Cu1	113.09 (18)	C16—C15—H15	118.8
C24—N3—C25	118.2 (2)	C17—C16—C15	119.6 (3)
C24—N3—Cu1	128.2 (2)	C17—C16—H16	120.2
C25—N3—Cu1	113.59 (18)	C15—C16—H16	120.2
C14—O4—Cu1^ii^	100.26 (16)	C16—C17—C18	119.5 (3)
C1—O1—Cu1	105.80 (18)	C16—C17—H17	120.2
C14—O3—Cu1^ii^	78.86 (16)	C18—C17—H17	120.2
O2—C1—O1	122.2 (3)	C26—C18—C17	116.8 (3)
O2—C1—C2	121.8 (3)	C26—C18—C19	118.6 (3)
O1—C1—C2	116.0 (3)	C17—C18—C19	124.5 (3)
C3—C2—C7	118.8 (3)	C20—C19—C18	120.5 (3)
C3—C2—C1	118.7 (3)	C20—C19—H19	119.7
C7—C2—C1	122.4 (3)	C18—C19—H19	119.7
C4—C3—C2	122.4 (3)	C19—C20—C21	122.1 (3)
C4—C3—H3	118.8	C19—C20—H20	118.9
C2—C3—H3	118.8	C21—C20—H20	118.9
C5—C4—C3	118.7 (3)	C25—C21—C22	116.2 (3)
C5—C4—H4	120.6	C25—C21—C20	118.3 (3)
C3—C4—H4	120.6	C22—C21—C20	125.5 (3)
C4—C5—C6	121.0 (3)	C23—C22—C21	120.2 (3)
C4—C5—H5	119.5	C23—C22—H22	119.9
C6—C5—H5	119.5	C21—C22—H22	119.9
C5—C6—C7	121.1 (3)	C22—C23—C24	119.8 (3)
C5—C6—H6	119.4	C22—C23—H23	120.1
C7—C6—H6	119.4	C24—C23—H23	120.1
N1—C7—C6	121.6 (3)	N3—C24—C23	122.0 (3)
N1—C7—C2	120.6 (2)	N3—C24—H24	119.0
C6—C7—C2	117.8 (3)	C23—C24—H24	119.0
C9—C8—C13	118.5 (3)	N3—C25—C21	123.6 (3)
C9—C8—N1	121.0 (3)	N3—C25—C26	116.4 (2)
C13—C8—N1	120.5 (2)	C21—C25—C26	120.1 (3)
C10—C9—C8	120.6 (3)	N2—C26—C18	123.8 (3)
C10—C9—H9	119.7	N2—C26—C25	115.8 (2)
C8—C9—H9	119.7	C18—C26—C25	120.3 (2)
			
O1—Cu1—N2—C15	142.7 (7)	C11—C12—C13—C14	−175.1 (3)
O4^i^—Cu1—N2—C15	−6.5 (3)	C9—C8—C13—C12	−0.3 (4)
N3—Cu1—N2—C15	179.8 (3)	N1—C8—C13—C12	−177.0 (3)
O3^i^—Cu1—N2—C15	−65.8 (3)	C9—C8—C13—C14	175.9 (3)
C14^i^—Cu1—N2—C15	−36.2 (3)	N1—C8—C13—C14	−0.8 (4)
O1—Cu1—N2—C26	−40.2 (8)	Cu1^ii^—O3—C14—O4	6.4 (2)
O4^i^—Cu1—N2—C26	170.57 (18)	Cu1^ii^—O3—C14—C13	−172.1 (3)
N3—Cu1—N2—C26	−3.10 (18)	Cu1^ii^—O4—C14—O3	−7.9 (3)
O3^i^—Cu1—N2—C26	111.35 (18)	Cu1^ii^—O4—C14—C13	170.6 (2)
C14^i^—Cu1—N2—C26	140.93 (18)	C12—C13—C14—O3	152.6 (3)
O1—Cu1—N3—C24	−1.7 (3)	C8—C13—C14—O3	−23.8 (4)
O4^i^—Cu1—N3—C24	130.9 (6)	C12—C13—C14—O4	−26.0 (4)
N2—Cu1—N3—C24	−177.4 (3)	C8—C13—C14—O4	157.7 (3)
O3^i^—Cu1—N3—C24	88.0 (3)	C12—C13—C14—Cu1^ii^	4.3 (10)
C14^i^—Cu1—N3—C24	89.9 (3)	C8—C13—C14—Cu1^ii^	−172.1 (7)
O1—Cu1—N3—C25	178.62 (19)	C26—N2—C15—C16	0.4 (4)
O4^i^—Cu1—N3—C25	−48.8 (7)	Cu1—N2—C15—C16	177.4 (2)
N2—Cu1—N3—C25	2.88 (18)	N2—C15—C16—C17	0.0 (5)
O3^i^—Cu1—N3—C25	−91.73 (19)	C15—C16—C17—C18	−0.4 (5)
C14^i^—Cu1—N3—C25	−89.8 (2)	C16—C17—C18—C26	0.3 (4)
O4^i^—Cu1—O1—C1	77.83 (18)	C16—C17—C18—C19	179.0 (3)
N3—Cu1—O1—C1	−108.10 (18)	C26—C18—C19—C20	0.6 (5)
N2—Cu1—O1—C1	−71.5 (8)	C17—C18—C19—C20	−178.1 (3)
O3^i^—Cu1—O1—C1	136.70 (18)	C18—C19—C20—C21	−1.3 (5)
C14^i^—Cu1—O1—C1	107.42 (18)	C19—C20—C21—C25	0.8 (5)
Cu1—O1—C1—O2	13.4 (3)	C19—C20—C21—C22	179.5 (3)
Cu1—O1—C1—C2	−167.57 (18)	C25—C21—C22—C23	0.2 (4)
O2—C1—C2—C3	160.8 (3)	C20—C21—C22—C23	−178.4 (3)
O1—C1—C2—C3	−18.2 (4)	C21—C22—C23—C24	−0.1 (5)
O2—C1—C2—C7	−19.0 (4)	C25—N3—C24—C23	0.7 (4)
O1—C1—C2—C7	162.0 (3)	Cu1—N3—C24—C23	−179.0 (2)
C7—C2—C3—C4	2.3 (4)	C22—C23—C24—N3	−0.4 (5)
C1—C2—C3—C4	−177.5 (3)	C24—N3—C25—C21	−0.5 (4)
C2—C3—C4—C5	1.8 (4)	Cu1—N3—C25—C21	179.2 (2)
C3—C4—C5—C6	−3.9 (5)	C24—N3—C25—C26	178.0 (3)
C4—C5—C6—C7	1.9 (5)	Cu1—N3—C25—C26	−2.2 (3)
C8—N1—C7—C6	29.3 (5)	C22—C21—C25—N3	0.1 (4)
C8—N1—C7—C2	−154.7 (3)	C20—C21—C25—N3	178.8 (3)
C5—C6—C7—N1	178.4 (3)	C22—C21—C25—C26	−178.4 (3)
C5—C6—C7—C2	2.3 (4)	C20—C21—C25—C26	0.3 (4)
C3—C2—C7—N1	179.6 (2)	C15—N2—C26—C18	−0.5 (4)
C1—C2—C7—N1	−0.6 (4)	Cu1—N2—C26—C18	−178.0 (2)
C3—C2—C7—C6	−4.2 (4)	C15—N2—C26—C25	−179.7 (3)
C1—C2—C7—C6	175.5 (2)	Cu1—N2—C26—C25	2.8 (3)
C7—N1—C8—C9	30.5 (5)	C17—C18—C26—N2	0.2 (4)
C7—N1—C8—C13	−152.8 (3)	C19—C18—C26—N2	−178.6 (3)
C13—C8—C9—C10	−1.3 (5)	C17—C18—C26—C25	179.3 (3)
N1—C8—C9—C10	175.4 (3)	C19—C18—C26—C25	0.6 (4)
C8—C9—C10—C11	2.0 (5)	N3—C25—C26—N2	−0.4 (4)
C9—C10—C11—C12	−1.0 (5)	C21—C25—C26—N2	178.2 (2)
C10—C11—C12—C13	−0.7 (5)	N3—C25—C26—C18	−179.6 (2)
C11—C12—C13—C8	1.3 (5)	C21—C25—C26—C18	−1.0 (4)

Symmetry codes: (i) *x*, −*y*+1, *z*−1/2; (ii) *x*, −*y*+1, *z*+1/2.

### Hydrogen-bond geometry (Å, º)

**Table:**

*D*—H···*A*	*D*—H	H···*A*	*D*···*A*	*D*—H···*A*
N1—H1···O2	0.86	2.07	2.708 (5)	131
N1—H1···O3	0.86	2.06	2.701 (5)	130
C17—H17···O2^iii^	0.93	2.55	3.308 (4)	139
C23—H23···O3^iv^	0.93	2.38	3.185 (4)	145

Symmetry codes: (iii) −*x*+1/2, −*y*+1/2, −*z*+1; (iv) −*x*+1/2, −*y*+3/2, −*z*+1.
